# BTNET : boosted tree based gene regulatory network inference algorithm using time-course measurement data

**DOI:** 10.1186/s12918-018-0547-0

**Published:** 2018-03-19

**Authors:** Sungjoon Park, Jung Min Kim, Wonho Shin, Sung Won Han, Minji Jeon, Hyun Jin Jang, Ik-Soon Jang, Jaewoo Kang

**Affiliations:** 1 Department of Computer Science and Engineering, Korea University, Seoul, Republic of Korea; 2 Interdisciplinary Graduate Program in Bioinformatics, Korea University, Seoul, Republic of Korea; 3 Division of Bioconvergence, Korea Basic Science Institute, Daejeon, Republic of Korea; 4 Genoplan Korea, Inc. and NAR Center, Inc., Seoul, Republic of Korea; 5 School of Industrial Management Engineering, Korea University, Seoul, Republic of Korea

**Keywords:** Gene regulatory network inference, Time course, Boosted tree

## Abstract

**Background:**

Identifying gene regulatory networks is an important task for understanding biological systems. Time-course measurement data became a valuable resource for inferring gene regulatory networks. Various methods have been presented for reconstructing the networks from time-course measurement data. However, existing methods have been validated on only a limited number of benchmark datasets, and rarely verified on real biological systems.

**Results:**

We first integrated benchmark time-course gene expression datasets from previous studies and reassessed the baseline methods. We observed that GENIE3-time, a tree-based ensemble method, achieved the best performance among the baselines. In this study, we introduce BTNET, a boosted tree based gene regulatory network inference algorithm which improves the state-of-the-art. We quantitatively validated BTNET on the integrated benchmark dataset. The AUROC and AUPR scores of BTNET were higher than those of the baselines. We also qualitatively validated the results of BTNET through an experiment on neuroblastoma cells treated with an antidepressant. The inferred regulatory network from BTNET showed that brachyury, a transcription factor, was regulated by fluoxetine, an antidepressant, which was verified by the expression of its downstream genes.

**Conclusions:**

We present BTENT that infers a GRN from time-course measurement data using boosting algorithms. Our model achieved the highest AUROC and AUPR scores on the integrated benchmark dataset. We further validated BTNET qualitatively through a wet-lab experiment and showed that BTNET can produce biologically meaningful results.

**Electronic supplementary material:**

The online version of this article (10.1186/s12918-018-0547-0) contains supplementary material, which is available to authorized users.

## Background

A gene regulatory network (GRN) is a biological network representing relationships between genes and their regulators. One representative regulator is a transcription factor that regulates a target gene’s expression. Reconstructing the gene regulatory network is important for understanding the biological system. The gene regulatory network could identify causal relationships among molecular interactions, help to prioritize experimental design, or be considered as network biomarkers [1]. Its applications are extended to elucidate disease processes [2] or to identify drug targets [3]. With the development of high-throughput technologies such as microarray and RNA-Seq [4, 5], gene expression data has become prevalent and a reliable source for reconstructing the gene regulatory network.

A good deal of research on reverse-engineering has been conducted using the gene expression data [6–9]. In the DREAM (Dialogue for Reverse Engineering Assessments and Methods) Challenges, methods were employed to construct a benchmark dataset that can be used to validate various inference algorithms [10, 11]. However, these methods rely on mostly steady-state expression data which is a snapshot of a biological process in a specific moment. To fully understand the dynamic properties of biological processes, it is essential to monitor their activity using time-course data [12]. Analyzing time-course data can help us to understand not only developmental and time-course biological processes but also mechanism of perturbation [1, 12].

Various GRN inference methods using time-course data have been developed [13–18]. Currently, the model-based and model-free approaches are the two main approaches. Model-based methods tend to formulate the expression of a target gene as a function of its regulators. Then, model-based methods use the learned parameters (coefficients) of regulators as regulatory interaction scores. Ridge regression, LASSO and Bayesian Model Averaging (BMA) are some of the representative methods of model-based methods [14, 15, 18, 19]. BGRMI, a recently developed GRN inference method, computes regulatory interaction scores using posterior probabilities obtained by BMA [18].

In contrast, model-free methods compute the degree of regulation based on information-theoretic criteria. TD-ARACNE [16] obtains time-delayed dependency between two genes by mutual information. Similarly, time-delayed ND [20] extracts dependencies based on cross-correlation instead and filters the indirect dependencies using network deconvolution method [21]. To deal with the dynamicity of regulatory delay induced by noisy environment, DDGni [22] captures the dynamic delay by applying the gapped local alignment algorithm.

One of the state-of-the-art methods used in model-free methods is GENIE3-time, a time-lagged version of GENIE3 [8, 13]. Basically, GENIE3 applies a tree-based ensemble method to compute scores of regulatory interactions. GENIE3 won both the DERAM4 *in-silico* multi-factorial challenge [10], and the DREAM5 network inference challenge [11] both in which various expression data were used for validating inference algorithms submitted by the participants of the challenges. GENIE3-time is an extended version of GENIE3 and used to infer networks from time-course expression data [13].

However, we found it difficult to objectively compare the performance of the current state-of-the-art methods because they were quantitatively validated on a small amount of dataset or different benchmark datasets. To address this problem, we integrated eight time-course gene expression benchmark datasets from the previous studies. Then, we re-evaluated the baseline methods [6, 8, 15, 17, 18, 20, 22, 23] on the integrated dataset. We found that GENIE3-time performed more robustly among the baseline methods (see Additional file 1: Table S1–S4).

In this article, we propose BTNET which is a boosted tree based gene regulatory network inference algorithm that is employed to reconstruct the network using time-course measurement data. The boosted tree is used to compute regulatory interaction scores between candidate regulators and target genes. To the best of our knowledge, this is the first study to use the boosted tree to infer GRNs using time-course measurement data. We evaluated BTNET on the integrated benchmark dataset and showed that our method outperformed 9 baselines including the current state-of-the-art method, GENIE3-time.

Furthermore, to verify if BTNET actually produces biologically meaningful networks, we qualitatively assessed the GRN inferred by BTNET using time-course data obtained from our experiments with antidepressant treated neuroblastoma cells. We treated SK-N-SH neuroblastoma cells with fluoxetine, an antidepresseant, and measured the transcription factors’ change in activity over time. From this data, BTNET inferred that brachyury, a transcription factor, was regulated by fluoxetine and this inference was validated by immunoblot assays.

## Methods

### Problem definition

In this section, we describe our inference model that reconstructs a gene regulatory network from time-course measurement data. Our model takes an *n* *T*×*P* expression matrix E as an input where *n* is the number of experiments, *T* is the number of times points and P is the total number of genes. Then, BTNET outputs a weighted adjacency matrix $W \in \mathbb {R}^{p \times p}$ where *w*_*i*,*j*_ is the regulatory interaction score that indicates how strongly gene *i* regulates gene *j*. We use only high confidence regulatory interactions where its scores are above the threshold to reconstruct a gene regulatory network.

### Tree-based ensemble method for inferring gene regulatory networks

The tree-based ensemble method, GENIE3 is one of the state-of-the-art approaches for inferring regulatory networks [8]. The method won the DERAM4 *in-silico* Multi-factorial Challenge [10], and DREAM5 Network Inference Challenge [11]. In GENIE3, the gene regulatory network inference problem is decomposed by *p* different subproblems where *p* denotes the number of genes in expression data. In each subproblem, one gene is considered as a target gene and other genes except the target gene are regarded as candidate regulators. Then, a bagging based ensemble tree method which is Random Forest [24] or Extra Tress [25] can compute the regulatory interaction scores between genes by measuring how strongly the expression values of candidate regulators contributed to predict expression values of a target gene. Computing the regulatory interaction scores and finding the regulators of a target gene can be viewed as a feature selection problem in machine learning.

GENIE3-time modifies GENIE3’s original regulatory interaction scoring method to compute the scores of candidate regulators for time-lagged expression value of a target gene [13]. Formally, *t*+1 time point expression values of a target gene are modeled by *t* time point values of candidate regulators as follows.

1 $$ e_{t+1}^{i} = f_{i}\left(e_{t}^{-i}\right) + \epsilon_{t}, \forall t,  $$

where $e_{t+1}^{i}$ represents the expression value of gene *i* at time point $t+1,e_{t}^{-i}$ the vector of expression value at time point *t* of genes except gene *i*, and *ε*_*t*_ indicates random noise at time t. A weighted adjacency matrix is then constructed after obtaining regulatory interaction scores of candidate regulators for each target gene *i* in total *P* genes. In a bagging procedure of GENIE3-time, regression trees are fitted to independent bootstrapped samples. Then, the ensemble score is obtained by averaging importance scores of all independently trained regression trees.

### BTNET

**Fig. 1:**
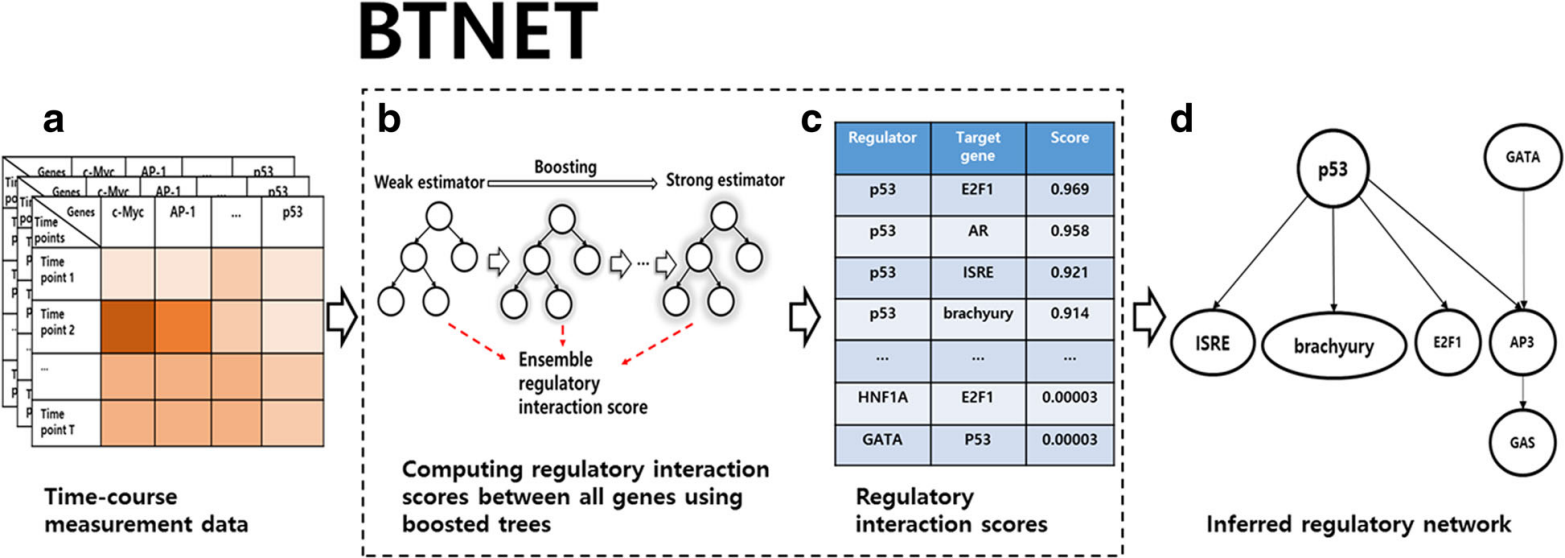
Overview of BTNET. **a** BTNET takes time-course measurement data as input. **b** Boosted tree (Adaboost or gradient boosting) is used to compute regulatory interaction scores for all pairs of genes. **c** BTNET outputs a weighted adjacency matrix which contains the regulatory interaction scores. **d** A gene regulatory network is reconstructed by only using high confidence regulatory interactions

In this article, we introduce a new ensemble tree based gene regulatory network inference algorithm that uses time-course measurement data when inferring the network. The ensemble tree we used for inferring the network is boosted tree. The boosted tree differs from bagging based tree applied in GENIE3-time in that while GENIE3-time aggregates multiple independent estimators for constructing the final ensemble method, boosted tree continuously updates estimator itself to make it stronger by compensating for the weakness of previous estimators [26]. We propose BTNET-AdaBoost and BTNET-GraBoost, both of which are based on popular tree-based boosting algorithms that use a regression tree as a base estimator, adaptive boosting (AdaBoost) and gradient boosting, respectively [27, 28]. The overview of BTNET is shown in Fig. 1. We first discuss each boosted tree algorithm and how we used the algorithms to compute regulatory interaction scores. Then, we will briefly describe the implementation and computational complexity of our method.

#### BTNET-AdaBoost

A brief explanation of the AdaBoost algorithm is given below. Let *f* be a base estimator, *T* the number of boosting iterations, *x*_*i*_ the feature vector of sample *i*, *N* the total number samples, and *L* be a loss function; then, AdaBoost is run using the following steps [27]

1. Assign initial sample weights where each sample *i* has a sample weight *w*_*i*_ where *w*_*i*_=1/*N*.
  This means all samples start with the same weight.
2. Build a training set size *N* by sampling with replacement according to the sample weights. The weight represents the probability of the samples being selected.
3. Train *f* on the sampled training set.
4. Make predictions on every training sample and compute normalized sample loss *err*_*i*_ by
  *err*_*i*_ = $\frac {|L(f(x_{i}),Y_{i})|}{\text {max}(|{L(f(x_{i}),Y_{i})}|)}$.
  Here, we use a linear loss function where
  *L*(*f*(*x*_*i*_),*Y*_*i*_)=(*Y*_*i*_−*f*(*x*_*i*_)).
5. Calculate average loss $\bar {L}$ with $\bar {L}$ = $\sum _{i=1}^{N}\ {err_{i}} {w}_{i}$.
6. Update the sample weights using *β* where
  *β* = $\frac {\bar {L}}{1-\bar {L}}$.
  The new sample weight is then, *w*_*i*_ = *w*_*i*_*β*^(1−*erri*)^.
7. Repeat steps 2 to 6 until boosting iteration becomes *T*.

Basically, a current estimator is fitted to “difficult” samples on which previous estimators obtained poor prediction performance. Prediction performance on difficult samples improved at the end of training. One characteristic of AdaBoost is that the weight of an estimator at each iteration can be obtained. The estimator weight is calculated as follows.

2 $$ {estimator\_weight}_{t} = {learning rate} \times {\text{log}{\frac{1}{\beta_{t}}}}  $$

where *t* indicates the stage of boosting from 1 to *T*.

Usually, the algorithm is used for solving prediction problems. However, for inferring regulatory network problems, we are more interested in what genes can be used to most accurately predict the expression values of target genes rather than how well the target gene expressions were predicted. The prediction accuracy is represented by the regulatory interaction score. To calculate it, we use variable importance scores from boosted tree that was trained to predict a target gene’s time-lagged expression from candidate regulator’s. The variable importance score of a single regression tree is calculated by how much a variable contributed to variance reduction after splitting training samples using the variable [29]. The Variable Importance Score (VIS) of gene *G* in one regression tree is calculated by the following equation.

3 $$ \begin{aligned} VIS(G) &= |S|Var(S) - |S_{left}|Var(S_{left})\\ &\quad- |S_{right}|Var(S_{right}) \end{aligned}  $$

where *S* is the set of samples in the current node and |*S*| refers to the size of *S*; *Var*(*S*) is the variance of the target values in the set *S*; *S*_*left*_ and *S*_*right*_ refer to the sets of samples in the left and right child nodes after splitting, respectively.

After obtaining VISs from all trees, the ensemble variable importance score is computed by aggregating of the scores. In AdaBoost, the ensemble importance score is calculated by the weighted average of VISs. Thus, the equation for computing ensemble VIS of a variable *G* is as follows.

4 $$ {{VIS}\_{ensemble}}(G) = \sum_{t=1}^{T}{estimator\_weight}_{t} \times {VIS_{t}}(G)  $$

By taking all the genes in the expression data as target genes and obtaining VISs for candidate regulators of the target genes, we could obtain regulatory interaction scores for all pairs of genes. The regulatory interaction scores are represented as a weighted adjacency matrix *W* where the value in *i*-th row and *j*-th column indicates the regulatory interaction score from gene *i* to gene *j*.

Once the adjacency matrix is obtained, only interactions that satisfy a certain threshold are represented by edges in the inferred gene regulatory network.

#### BTNET-GraBoost

We use gradient boosting, another boosted tree based ensemble inference method, for scoring regulatory interactions. Gradient boosting was also successfully used for inferring gene regulatory networks from steady-state gene expression data [9, 30]. The gradient boosting algorithm follows the gradient descent procedure that is employed to minimize the loss *L* of an estimator *f* by adding residual fitted estimator *h* [28]. The loss function *L* used here is based on squared error as follows.

5 $$ L\left(f(x_{i}),Y_{i}\right) = \frac{(Y_{i}-f(x_{i}))^{2}}{2}  $$

Then, residual *R* is obtained by derivative of *L* by *f*.

6 $$ R\left(f(x_{i}),Y_{i}\right) = \frac{\partial L\left(f(x_{i}),Y_{i}\right)}{\partial f(x_{i})} = Y_{i} - f(x_{i})  $$

where *f*(*x*_*i*_) denotes a prediction value of *i*-th sample and *Y* denotes a target value of the *i*-th sample. In Gradient boosting, a base estimator *f*_0_ produces its prediction by simply averaging the target values.

7 $$ f_{0} = \bar{Y}  $$

At each stage *t*, a new estimator *h*_*t*_ is fitted to the residuals *R* of previous estimator *f*_*t*−1_ where the residuals are derivatives of square loss function *L* over *f*_*t*−1_. Then, *h*_*t*_ is added to the previous learner with the learning rate *β*.

8 $$ f_{t} = f_{t-1} + \beta{h_{t}}  $$

The additive estimator *f*_*t*_ continuously improve its prediction power by compensating the previous estimator’s error.

The only difference between BTNET-AdaBoost and BTNET-GraBoost, other than the boosting method itself (i.e., AdaBoost vs gradient boosting), comes from aggregating method of single trees’ variable importance scores. In the case of BTNET-AdaBoost, the ensemble importance scores were computed by weighted average whereas the ensemble scores of BTNET-GraBoost were obtained by just averaging the importance scores of each tree’s as follows.

9 $$ {{VIS}\_{ensemble}}(G) = \frac{1}{T}\sum_{t=1}^{T}{VIS_{t}}(G)  $$

The methods for computing variable importance scores in a single tree, obtaining the weighted adjacency matrix that contains regulatory interaction scores for all gene pairs, and constructing a GRN are the same as those used in BTNET-Adaboost.

#### Implementation

We built BTNET by modifying GENIE3-time python implementation [13]. We modified the part of computing regulatory interaction scores from bagging based tree method to boosted tree method. We used AdaBoost and gradient boosting implementation provided in the scikit-learn Python machine learning package. The visualization of an inferred gene regulatory network was done by using the Graphviz Python package version 0.4.10. For a fair comparison with GENIE3-time, we used the same parameter conditions as GENIE3-time from Jump3 [17] on both BTNET-AdaBoost and BTNET-GraBoost (n_estimators as 100, and others as default values of scikit-learn package). All genes except for a target gene were regarded as candidate regulators.

### Computational complexity

The computational complexity of BTNET is the same as that of GENIE3-time, which is *O*(*pTKNlogN*) where *p* is the number of genes, *K* is the number of candidate regulators, *T* is the number of iterations for boosting, and *N* is the total number of samples. Training one regression tree has a complexity on the order of *O*(*KNlogN*). Since building an ensemble tree takes *T* times longer than a single tree, both BTNET-AdaBoost and BTNET-GraBoost require a time complexity on the order of *O*(*TKNlogN*). To obtain full regulatory networks, the ensemble tree must be fit to *p* total genes. Therefore, BTNET has a computational complexity on the order of *O*(*pTKNlogN*).

## Results and discussion

In this section, we briefly describe the 8 benchmark datasets we used for the quantitative evaluations and report the AUROC and AUPR scores of our BTNET method and 9 baseline methods. We also report the results of qualitative analysis that experimentally verifies a regulatory interaction inferred by BTNET using antidepressant treated human SK-N-SH neuroblastoma cells.

### Benchmark datasets

#### IRMA dataset

The in vivo reverse-engineering and modeling assessment (IRMA) network is a yeast (Saccharomyces cerevisiae) synthetic network that was made for validating the performance of GRN inference methods [31]. The network consists of 5 genes (CBF1, GAL4, SWI5, GAL80 and ASH1). The original IRMA network has 7 regulatory interactions (CBF1 - > GAL4, GAL4 - > SWI5, GAL4 - > GAL80, SWI5 - > ASH1, SWI5 - > GAL80, ASH1 - > CBF1, and GAL80 - > GAL4). In the simplified version, an interaction from GAL40 to GAL4 was omitted. Switch-on and switch-off data, two types of time-course gene expression data, are from the IRMA network. In switch-on data, 16 time points of gene expressions were measured after the IRMA network was activated by galactose. In switch-off data, 21 time points of expressions were measured after switching the galactose to glucose. We inferred a network from switch-on and another network from switch-off data, and evaluated the networks against the original network and simplified network, respectively.

#### Spellman dataset

The Spellman dataset contains time-course gene expression data on yeast (Saccharomyces cerevisiae) cell cycle [32]. We selected two types of expression dataset which were cdc-15 dataset, and cdc-28 dataset. Cdc-15 and cdc-28 dataset were made by measuring 24 and 17 time points expressions of 9 genes (FKH2, SWI4, SWI5, SWI6, NDD1, ACE2, CLN3, MBP1, and MCM1) from cdc-15 and cdc-28 cell cycle arrested yeast, respectively. Yeast cell cycle network used for the ground truth network of the Spellman dataset was obtained from the study by Simon et al. (2001) [33].

#### C.elegans and yeast cell cycle data from DDGni

We obtained time-course gene expression dataset of Caenorhabditis elegans (C.elegans) and yeast cell cycle, and the ground truth networks for each dataset from the study named DDGni [22]. The C.elegans dataset contains 6 genes (PHA-4, END-1, ELT-2, ELT-7, GES-1, and END-3) and 180 time points of gene expressions were measured using cell imaging techniques [34]. The ground truth networks were manually constructed by the authors of DDGni. In case of yeast cell cycle dataset, the authors of DDGni obtained the dataset from GEO [35] with the accession number GSE8799. They selected 8 well-researched TFs which are YOX1, STB1, HCM1, WHI5, YHP1, ACE2, SWI5 and ASH1 [36] from the GEO dataset and manually constructed a ground truth network from the literature and external databases (YEASTACT [37] and STRING [38]). The yeast cell cycle time-course dataset contains 30 time points of expression values.

#### DREAM4 *in silico* dataset

DREAM4 *in silico* time-course dataset, from the DREAM4 *In Silico* Network Challenge, is well-known simulated benchmark dataset used for assessing network inference methods. Among 10 networks that were provided in the challenge, five networks had 10 genes and the other five had 100 genes. The networks containing 10 genes and the others containing 100 genes have 5 and 10 replicates of time-course expression data, respectively. Each replicate has 21 time points. At t=0, about one third of the genes were perturbed by increasing or decreasing initial expression of those genes. After 10 time points, the perturbation is then removed and returned to its original state. Initially perturbed genes were different from each replicate.

### Performance metrics

The output of BTNET is a weighted adjacency matrix containing regulatory interaction scores of all possible interactions between all genes. To form a gene regulatory network, we select a subset of interactions where the regulatory interaction scores are above a certain threshold. Area Under Receiver Operating Characteristic (AUROC) and Area Under Precision-Recall (AUPR) have usually been used to evaluate the performance of GRN inference methods [8, 10, 11, 17, 18]. AUROC calculates the area under a ROC (receiver operating characteristic) curve where the x-axis indicates a false positive rate (FPR) and the y-axis indicates a true positive rate (TPR). In the case of AUPR, it calculates the area under a precision-recall curve where the x-axis indicates recall and the y-axis indicates precision.

### Performance on benchmark datasets

We conducted a quantitative evaluation of BTNET by comparing AUROC and AUPR scores on the eight benchmark datasets. On the IRMA dataset, we inferred two GRNs from switch-on and switch-off time-course data. We inferred two GRNs from Spellman cdc-15 and cdc-28 time-course data. We also obtained two GRNs from C.elegans and yeast cell cycle time-course data. On DREAM4 dataset, we inferred five GRNS for each DREAM4 *in silico*-size10 and DREAM4-size100 dataset having 10 GRNs in total. In case of the DREAM4 dataset, we averaged AUROC/AUPR scores for each five networks of the size10 and size100 networks, respectively. Thus, we received 10 evaluation results for each AUROC and AUPR scores. The difference between the number of datasets and evaluations in the IRMA dataset was caused by evaluating two inferred GRNs from switch-on and switch-off data with the two ground truth networks (original IRMA and simplified IRMA networks) producing four evaluation results. We averaged the 10 scores for each AUROC and AUPR and compared BTNET with the following nine baseline methods: BGRMI [18], JUMP3 [17], GENIE3-time using Random Forest and Extra Trees [8], DDGni [22], TDARACNE [16], TSNI [23], timedelayND [20], time-lagged clr [6] and inferelator [15]. As shown in Table 1, our BTNET method achieved better AUPR scores than all baseline methods. BTNET-GraBoost achieved the highest on both AUPR and AUROC scores. BTNET-GraBoost also presented lower standard deviations of AUROC and AUPR scores in comparison to GENIE3-time. Furthermore, BTNET-GraBoost showed the best results in average ranks on both AUROC and AUPR scores. Results indicate that our methods are not only more accurate but also more robust than baseline methods. All AUROC/AUPR scores and ranks for each dataset are in Additional file 1 (see Table S1–S4).

**Table 1:** Overall Scores of AUROC and AUPR on the integrated benchmark dataset

	AUPR			AUROC		
	Avg	Std	Avg rank	Avg	Std	Avg rank
BTNET-GB	0.453	0.216	3.7	0.668	0.108	3.6
BTNET-AB	0.445	0.237	4.3	0.645	0.142	4.7
GENIE3-time_RF	0.43	0.227	4.3	0.652	0.142	3.8
BGRMI	0.419	0.304	5.4	0.596	0.261	5.6
Jump3	0.397	0.244	5.1	0.63	0.113	5.5
Inferelator	0.39	0.27	5.6	0.611	0.111	5.3
GENIE3-time_ET	0.381	0.2	5.7	0.606	0.153	6
DDGni	0.346	0.217	7.75	0.621	0.099	7.125
CLR-lag	0.344	0.212	7	0.563	0.17	7.1
TSNI	0.343	0.226	7	0.564	0.142	7
time-delayed ND	0.259	0.165	9.5	0.476	0.122	9.3

### Qualitative analysis of BTNET on antidepressant treated human neuroblastoma cells

To further evaluate BTNET, we performed an additional qualitative analysis using wet lab experiments. We first inferred a regulatory network from time-course activity data of transcription factors using BTNET. The activity of transcription factors was measured after treating human neuroblastoma cell line SK-N-SH with fluoxetine, a popular antidepressant. Twenty depression related transcription factors (MEF1, AP-3, HNF1A, ARNT1, GAS, AP-4, GATA, c-Myc, brachyury, ER, AP-2, LHX8, ETS1, AP-1, AR, p53, E2F1, FOXC2, HSE and ISRE) were chosen for measuring activities. Living cell array [39] was used to measure the activities of the transcription factors. The activities were measured every 12 h for 10 days with 3 replicates. Thus, three 20 × 20 time-course input matrices were used for inference. A detailed description on the materials and methods used for this experiment is provided in Additional file 1 (see Materials and methods of qualitative analysis).

**Fig. 2:**
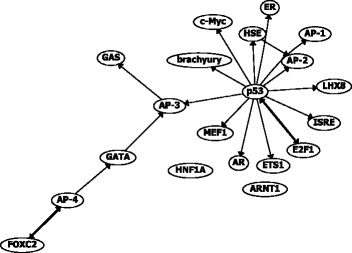
Regulatory network inferred by BTNET using time-course data of fluoxetine-treated SK-N-H cells

The inferred network is shown in Fig. 2. BTNET-GraBoost was used to infer the network. We used 0.25 as the threshold for the regulatory interaction score as it exhibited the best F1-score on the 10 benchmark evaluations. The network shows regulatory relationships between 20 TFs affected by fluoxetine, and indicates that p53 acts as a central regulator of the fluoxetine-induced network. Previous studies have identified the effects of fluoxetine on p53 [40, 41].

Additionally, Fig. 2 indicates that fluoxetine regulated the activity of brachyury via p53. Brachyury is a transcription factor and its main function is promoting epithelial-mesenchymal transition (EMT) by down-regulating E-cadherin [42, 43]. Studies reported that brachyury is also involved in several types of tumors [44, 45]. In particular, It was reported that brachyury is a biomarker for chordomas, which is a type of central nervous system tumor [44]. However, the inferred relation that fluoxetine regulates brachyury via p53 is novel and has not been reported before.

**Fig. 3:**
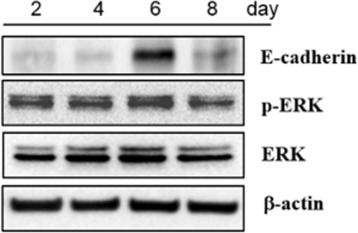
Immunoblot assay result showing increased expression of downstream molecules of brachyury

To verify the inferred relation, immunoblot assay was examined. In Fig. 3, the immunoblot assay shows expression levels of downstream molecules of brachyury, such as E-cadherin [46] and p-ERK [47], were elevated (day 6). ERK and *β*-actin were used as controls for protein quantification. The immunoblot assay result demonstrates that brachyury was in fact regulated by fluoxetine, and fluoxetine may have affected brachyury between day 4 and day 6 after the treatment. Additional file 1: Figure S1 shows the activities of p53 and brachyury measured in the living cell array. It shows that brachyury was actually upregulated between day 5 and day 6.

## Conclusions

We developed a more accurate and robust method that infers GRNs from time-course measurement data. Most GRN methods using time-course data were validated only on a limited number of benchmark datasets. To address this problem, we integrated time-course gene expression datasets from previous studies and re-evaluated the baseline methods on the integrated benchmark set. GENIE3-time achieved the best performance among the baseline methods. GENIE3-time infers GRNs by computing all possible pairs of regulators-target gene regulatory interaction scores using Random Forest (or Extra Trees). We attempted to improve the current state-of-the-art method, GENIE3-time, by using boosting algorithms to compute regulatory interaction scores.

We proposed two boosted tree based GRN inference methods: BTNET-AdaBoost and BTNET-GraBoost. BTNET-AdaBoost uses adaptive boosting and BTNET-GraBoost uses gradient boosting to compute the regulatory interaction scores. BTNET-GraBoost achieved the highest AUPR/AUROC scores and the best average ranks. We performed wet lab experiments to validate whether BTNET could infer biologically meaningful networks. Living cell array analysis was used to analyze the activity of TFs in real time at various time points after treating human SK-N-SH cell lines with fluoxetine. BTNET inferred a regulatory network from the time-course data and brachyury was shown to be regulated by fluoxetine. The inferred regulation of brachyury was verified by testing the expression of downstream molecules of the TF and actual increase of expression on brachyury’s downstream molecules was observed.

## Additional file

Additional file 1 Supplementary file of BTNET. The file contains the URL of the source code and dataset used in this study, evaluation results on individual benchmark datasets and materials and methods used for the qualitative analysis. (PDF 263 kb)
