# Supplementary material for: BTNET : boosted tree based gene regulatory network inference algorithm using time-course measurement data
Source: BMC Syst Biol. 2018 Mar 19;12(Suppl 2):20. doi: 10.1186/s12918-018-0547-0 (PMC5861501; doi:10.1186/s12918-018-0547-0)
Supplement: Supplementary file 1 — Supplementary file of BTNET. The file contains the URL of the source code and dataset used in this study, evaluation results on individual benchmark datasets and materials and methods used for the qualitative analysis. (PDF 263 kb) [file 12918_2018_547_MOESM1_ESM.pdf]

# Supplementary file of BTNET

Sungjoon Park[1,†], Jung Min Kim[4,†], Wonho Shin[2], Sung Won Han[5], Minji Jeon[1], Hyun Jin Jang[3], Ik-Soon Jang[3,*], and Jaewoo Kang[1,2,*]

[1]Department of Computer Science and Engineering, Korea University, Seoul, Republic of Korea
[2]Interdisciplinary Graduate Program in Bioinformatics, Korea University, Seoul, Republic of Korea
[3]Division of Bioconvergence, Korea Basic Science Institute, Daejeon, Republic of Korea
[4]Genoplan Korea, Inc. and NAR Center, Inc., Seoul, Republic of Korea
[5]School of Industrial Management Engineering, Korea University, Seoul, Republic of Korea

## 1 Codes and Data

The source codes and all the time-course datasets used in this study can be downloaded from infos.korea.ac.kr/BTNET

## 2 Evaluation results on benchmark datasets

Table S1: AUPR results on benchmark datasets

| | IRMA-On | IRMA-Off | IRMA(S)-On | IRMA(S)-Off | DDGni-E | DDGni-Yeast | Dream4-size10 | Dream4-size100 | Spellman cdc15 | Spellman cdc28 | Avg | Std |
|---|---|---|---|---|---|---|---|---|---|---|---|---|
| BTNET-GB | 0.802 | 0.637 | 0.647 | 0.59 | 0.332 | 0.372 | 0.532 | 0.171 | 0.242 | 0.208 | 0.453 | 0.216 |
| BTNET-AB | 0.715 | 0.803 | 0.632 | 0.539 | 0.171 | 0.468 | 0.518 | 0.174 | 0.226 | 0.202 | 0.445 | 0.237 |
| Genie3-RF | 0.826 | 0.63 | 0.603 | 0.496 | 0.181 | 0.421 | 0.546 | 0.183 | 0.209 | 0.205 | 0.43 | 0.227 |
| BGRMI | 0.806 | 0.505 | 0.906 | 0.574 | 0 | 0.209 | 0.618 | 0.184 | 0.202 | 0.188 | 0.419 | 0.304 |
| JUMP3 | 0.747 | 0.714 | 0.36 | 0.682 | 0.179 | 0.147 | 0.513 | 0.187 | 0.259 | 0.189 | 0.397 | 0.244 |
| Inferelator | 0.833 | 0.771 | 0.682 | 0.315 | 0.17 | 0.27 | 0.335 | 0.114 | 0.111 | 0.298 | 0.39 | 0.27 |
| Genie3-ET | 0.656 | 0.667 | 0.522 | 0.261 | 0.174 | 0.424 | 0.521 | 0.175 | 0.215 | 0.192 | 0.381 | 0.2 |
| DDGni | 0.626 | 0.662 | 0.459 | 0.254 | 0.206 | 0.295 | 0.234 | 0.034 | | | 0.346 | 0.217 |
| CLR-lag | 0.726 | 0.373 | 0.63 | 0.325 | 0.124 | 0.375 | 0.398 | 0.051 | 0.165 | 0.274 | 0.344 | 0.212 |
| TSNI | 0.634 | 0.491 | 0.708 | 0.514 | 0.154 | 0.285 | 0.206 | 0.043 | 0.174 | 0.218 | 0.343 | 0.226 |
| timedelayND | 0.579 | 0.453 | 0.347 | 0.317 | 0.136 | 0.168 | 0.183 | 0.021 | 0.182 | 0.201 | 0.259 | 0.165 |

Table S2: AUROC results on benchmark datasets

| | IRMA-On | IRMA-Off | IRMA(S)-On | IRMA(S)-Off | DDGni-E | DDGni-Yeast | Dream4-size10 | Dream4-100 | Spellman cdc15 | Spellman cdc28 | Avg | Std |
|---|---|---|---|---|---|---|---|---|---|---|---|---|
| BTNET-GB | 0.806 | 0.71 | 0.719 | 0.704 | 0.498 | 0.655 | 0.767 | 0.741 | 0.584 | 0.495 | 0.668 | 0.108 |
| BTNET-AB | 0.681 | 0.781 | 0.621 | 0.669 | 0.391 | 0.785 | 0.775 | 0.75 | 0.55 | 0.444 | 0.645 | 0.142 |
| Genie3-RF | 0.793 | 0.712 | 0.671 | 0.621 | 0.44 | 0.793 | 0.775 | 0.764 | 0.511 | 0.44 | 0.652 | 0.142 |
| BGRMI | 0.781 | 0.572 | 0.916 | 0.559 | 0 | 0.8 | 0.736 | 0.706 | 0.467 | 0.424 | 0.596 | 0.261 |
| JUMP3 | 0.781 | 0.687 | 0.666 | 0.523 | 0.465 | 0.64 | 0.728 | 0.742 | 0.6 | 0.464 | 0.63 | 0.113 |
| Inferelator | 0.781 | 0.718 | 0.678 | 0.535 | 0.465 | 0.695 | 0.664 | 0.596 | 0.464 | 0.517 | 0.611 | 0.111 |
| Genie3-ET | 0.662 | 0.656 | 0.561 | 0.464 | 0.42 | 0.828 | 0.769 | 0.774 | 0.51 | 0.412 | 0.606 | 0.153 |
| DDGni | 0.744 | 0.63 | 0.636 | 0.434 | 0.6 | 0.75 | 0.591 | 0.585 | | | 0.621 | 0.099 |
| CLR-lag | 0.703 | 0.447 | 0.702 | 0.494 | 0.232 | 0.768 | 0.694 | 0.622 | 0.383 | 0.586 | 0.563 | 0.17 |
| TSNI | 0.604 | 0.468 | 0.785 | 0.619 | 0.375 | 0.796 | 0.527 | 0.559 | 0.4 | 0.508 | 0.564 | 0.142 |
| timedelayND | 0.609 | 0.479 | 0.458 | 0.333 | 0.27 | 0.703 | 0.495 | 0.488 | 0.438 | 0.484 | 0.476 | 0.122 |

*We couldn't run DDGni on the Spellman datasets since DDGni requires input data that doesn't contain zero values.

Table S3: AUPR ranks on benchmark datasets

| | IRMA-On | IRMA-Off | IRMA(S)-On | IRMA(S)-Off | DDGni-E | DDGni-Yeast | Dream4-size10 | Dream4-100 | Spellman cdc15 | Spellman cdc28 | Avg |
|---|---|---|---|---|---|---|---|---|---|---|---|
| BTNET-GB | 4 | 6 | 4 | 2 | 1 | 5 | 3 | 6 | 2 | 4 | 3.7 |
| BTNET-AB | 7 | 1 | 5 | 4 | 6 | 1 | 5 | 5 | 3 | 6 | 4.3 |
| Genie3-RF | 2 | 7 | 7 | 6 | 3 | 3 | 2 | 3 | 5 | 5 | 4.3 |
| BGRMI | 3 | 8 | 1 | 3 | 11 | 9 | 1 | 2 | 6 | 10 | 5.4 |
| JUMP3 | 5 | 3 | 10 | 1 | 4 | 11 | 6 | 1 | 1 | 9 | 5.1 |
| Inferelator | 1 | 2 | 3 | 9 | 7 | 8 | 8 | 7 | 10 | 1 | 5.6 |
| Genie3-ET | 8 | 4 | 8 | 10 | 5 | 2 | 4 | 4 | 4 | 8 | 5.7 |
| DDGni | 10 | 5 | 9 | 11 | 2 | 6 | 9 | 10 | | | 7.75 |
| CLR-lag | 6 | 11 | 6 | 7 | 10 | 4 | 7 | 8 | 9 | 2 | 7 |
| TSNI | 9 | 9 | 2 | 5 | 8 | 7 | 10 | 9 | 8 | 3 | 7 |
| timedelayND | 11 | 10 | 11 | 8 | 9 | 10 | 11 | 11 | 7 | 7 | 9.5 |

Table S4: AUROC ranks on benchmark datasets

| | IRMA-On | IRMA-Off | IRMA(S)-On | IRMA(S)-Off | DDGni-E | DDGni-Yeast | Dream4-size10 | Dream4-100 | Spellman cdc15 | Spellman cdc28 | Avg |
|---|---|---|---|---|---|---|---|---|---|---|---|
| BTNET-GB | 1 | 4 | 3 | 1 | 2 | 10 | 4 | 5 | 2 | 4 | 3.6 |
| BTNET-AB | 8 | 1 | 9 | 2 | 7 | 5 | 2 | 3 | 3 | 7 | 4.7 |
| Genie3-RF | 2 | 3 | 6 | 3 | 5 | 4 | 1 | 2 | 4 | 8 | 3.8 |
| BGRMI | 3 | 8 | 1 | 5 | 11 | 2 | 5 | 6 | 6 | 9 | 5.6 |
| JUMP3 | 5 | 5 | 7 | 7 | 3 | 11 | 6 | 4 | 1 | 6 | 5.5 |
| Inferelator | 3 | 2 | 5 | 6 | 3 | 9 | 8 | 8 | 7 | 2 | 5.3 |
| Genie3-ET | 9 | 6 | 10 | 9 | 6 | 1 | 3 | 1 | 5 | 10 | 6 |
| DDGni | 6 | 7 | 8 | 10 | 1 | 7 | 9 | 9 | | | 7.125 |
| CLR-lag | 7 | 11 | 4 | 8 | 10 | 6 | 7 | 7 | 10 | 1 | 7.1 |
| TSNI | 11 | 10 | 2 | 4 | 8 | 3 | 10 | 10 | 9 | 3 | 7 |
| timedelayND | 10 | 9 | 11 | 11 | 9 | 8 | 11 | 11 | 8 | 5 | 9.3 |

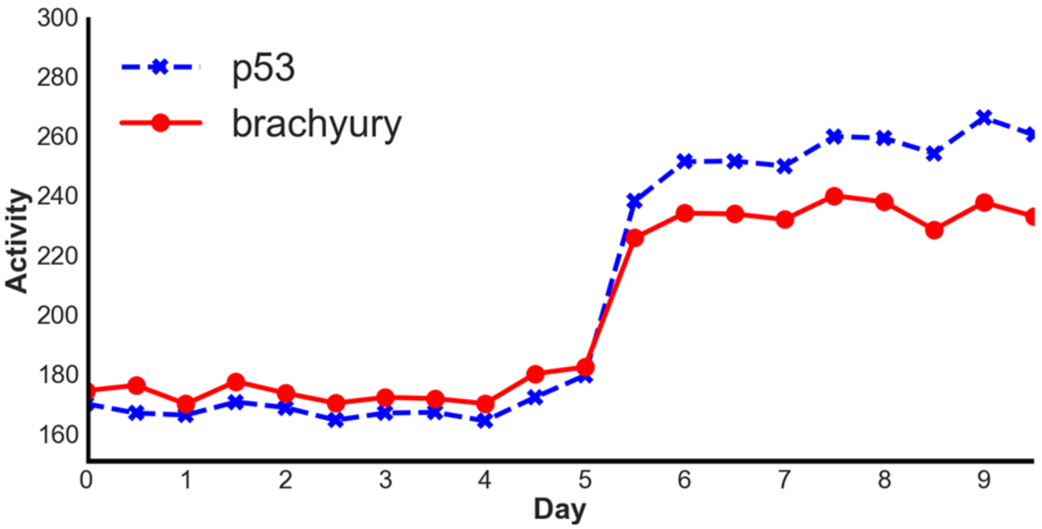


Figure S1: Line graph showing activities of p53 and brachyury measured in the living cell array

# 3 Materials and methods of qualitative analysis

## 3.1 Cell culture

Human SK-N-SH neuroblastoma cells were obtained from American Type Culture Collection (Rockville, MD, USA). Cells were cultured in DMEM medium containing 10% FBS (PAN biotech, Aidenbach, Germany) and 1% Antibiotics-antimycotics (Thermo Scientific, San Jose, CA, USA ) solution. Every 2 3 days, medium were changed and passaged by trypsinization. The cells were incubated in a 5% CO2 incubator at 37°C.

## 3.2 Vector cloning

20 transcription factor (TF) binding site encoding sequences were amplified by PCR. PCR products were gel purified and inserted into pGreenFire1$^{TM}$-mCMV-EF1-Puro vector (System Biosciences,

CA, USA) The double-stranded DNA fragments digested by restriction enzymes(EcoRI and SpeI; NEB, MA, USA) were ligated to the linearized lentivirus vector with T4 ligase. The ligated products were transformed into E.coli DH5-$\alpha$ cells (Enzynomics, Daejeon, Korea), and selected by sequencing verification.

### 3.3 Virus packaging and Virus transduction

Human 293T embryonic kidney cells were seeded in a 75cm2 cell culture flask (3 x 106 cells per culture dish) with DMEM containing 10% FBS. The expression vector and lentivirus packaging mixture (Invitrogen, Carlsbad, CA, USA) were transfected into 293T cells with Hillymax (Dojindo, Kumamoto, Japan) and opti-MEM medium. After 5 hours of transfection, the primary mixture was replaced with 1.0 g/L glucose DMEM medium with 5% FBS and supernatant were collected 48 hours after transfection. The collected supernatant was filtered by 0.2 um pore size syringe filter and stored at -8°C.

### 3.4 Live cell analysis for expression of transcription factors

The SK-N-SH cells were seeded on collagen coated 96 well plate. After 24 hours from seeding, the cells were transduced using each virus supernatant for 48 hours before treatment of antidepressant fluoxetine (Sigma-Aldrich ,St. Louis, MO, USA). And then, the fluoxetine was treated indicated concentration during 10 days. Fluorescence was evaluated every 12 hours. Biofluorescence analysis was performed with a multiple plate reader (TriStar$^2$ S LB 942, Berthold technologies, Bad Wildbad, Germany). Normalized light emission was determined by blank group and normalized transcription factor activity was evaluated by non-treated group.

### 3.5 Immunoblot assay

Cells were washed with PBS and then, followed by scraping on ice in Mammalian protein extraction buffer (GE healthcare, Chicago, USA) phosphatase cocktail 2, phosphatase cocktail 3, and protease inhibitor (Sigma-Aldrich). After freezing and thawing with liquid nitrogen twice, the lysate was centrifuged (16200 rpm for 15 min at 4 °C), and protein concentration of the supernatant was measured. The protein content was quantified by a Pierece BCA protein assay kit (Thermo Scientific). Total protein was run on 10% SDS–polyacrylamide gels and the proteins were electroblotted onto PVDF membranes (Bio-Rad, Hercules, CA, USA) and then were probed using E-cadherin, ERK1/2 (Cell Signaling Technology, Beverly, MA, USA) and $\beta$-actin, p-ERK (Santa Cruz, Dallas, TX, USA). The protein bands were detected by enhanced chemiluminescence (Thermo Scientific) and densitometric measurements of the bands were performed using Image J software.

## References

Bansal, M., Della Gatta, G., and Di Bernardo, D. (2006). Inference of gene regulatory networks and compound mode of action from time course gene expression profiles. *Bioinformatics*, **22**(7), 815–822.

Bonneau, R., Reiss, D. J., Shannon, P., Facciotti, M., Hood, L., Baliga, N. S., and Thorsson, V. (2006). The inferelator: an algorithm for learning parsimonious regulatory networks from systems-biology data sets de novo. *Genome biology*, **7**(5), R36.

Chen, H., Mundra, P. A., Zhao, L. N., Lin, F., and Zheng, J. (2014). Highly sensitive inference of time-delayed gene regulation by network deconvolution. *BMC systems biology*, **8**(4), S6.

Huynh-Thu, V. A. (2012). *Machine learning-based feature ranking: statistical interpretation and gene network inference*. Ph.D. thesis, Université de Liège, Liège, Belgium.

Iglesias-Martinez, L. F., Kolch, W., and Santra, T. (2016). Bgrmi: A method for inferring gene regulatory networks from time-course gene expression data and its application in breast cancer research. *Scientific Reports*, **6**.

Irrthum, A., Wehenkel, L., Geurts, P., *et al.* (2010). Inferring regulatory networks from expression data using tree-based methods. *PloS one*, **5**(9), e12776.

Sanguinetti, G. *et al.* (2015). Combining tree-based and dynamical systems for the inference of gene regulatory networks. *Bioinformatics*, page btu863.

Yalamanchili, H. K., Yan, B., Li, M. J., Qin, J., Zhao, Z., Chin, F. Y., and Wang, J. (2013). Ddgni: Dynamic delay gene-network inference from high-temporal data using gapped local alignment. *Bioinformatics*, page btt692.
